# Ten-year epidemiological study of ocular and orbital tumors in Chiba University Hospital

**DOI:** 10.1186/s12886-021-02108-w

**Published:** 2021-09-23

**Authors:** Norihiro Shimizu, Toshiyuki Oshitari, Jiro Yotsukura, Hirotaka Yokouchi, Takayuki Baba, Shuichi Yamamoto

**Affiliations:** 1 Department of Ophthalmology and Visual Science, Chiba University Graduate School of Medicine, Inohana 1-8-1, Chuo-ku, Chiba, 260-8670 Japan; 2 Department of Ophthalmology, International University of Health and Welfare, School of Medicine, 4-3 Kozunomori, Narita, Chiba, 286-8686 Japan

**Keywords:** Orbital tumor, Eyelid tumor, Malignant lymphoma, IgG4-related diseases, Histopathology

## Abstract

**Background:**

The purpose of this study is to determine the epidemiology of tumors of the ocular adnexa and orbit in Japan.

**Methods:**

We conducted a retrospective study on the histopathological reports in the medical records of the Chiba University Hospital from April 2009 to March 2019. Three hundred and seventy two records were examined. In addition, we examined the annual changes in the major types of tumors including malignant lymphomas and IgG4-related diseases (IgG4-RDs).

**Results:**

There were 270 conjunctival or eyelid tumors with 166 benign and 104 malignant. There were 102 orbital tumors with 55 benign, 47 malignant tumors, and 21 cases of IgG4-RDs. Ten cases of adenoma (2.7%), another benign tumor, was also diagnosed. The major malignant tumors were malignant lymphoma in 74 cases, sebaceous gland carcinoma (SGC) in 28 cases, basal cell carcinoma in 15 cases, and squamous cell carcinoma in 8 cases. The SGCs were the most common malignant eyelid tumor at 54%. Among the malignant lymphomas, extranodal marginal zone lymphomas of the mucosa-associated lymphoid tissue type, MALT lymphomas, was the most common at 51 cases and the second most common was the diffuse large B-cell lymphoma at 11 cases. The ratio of MALT lymphomas to that of all malignant lymphomas increased significantly with years. The serum IgG4 values were measured more often in the last 5 years (70%) than in the former 5 years (33%).

**Conclusions:**

We conclude that malignant lymphoma is a major malignant tumor in Japan and pathological biopsies should be done proactively to prevent missing IgG4-positive MALT lymphomas.

**Supplementary Information:**

The online version contains supplementary material available at 10.1186/s12886-021-02108-w.

## Background

Ocular adnexa and orbital tumors are generally difficult to diagnose without histopathological examinations, and many are malignant and life-threatening. Patients who have ocular adnexa and orbital tumors need to be examined histopathologically to make an accurate diagnosis but these examinations are difficult to perform in private clinics and hospitals without specialists. In fact, most patients with ocular adnexa and orbital tumors in Chiba prefecture with a population of 6.28 million are referred to the Chiba University Hospital. These types of tumors are relatively rare compared to other ocular disorders, thus most ophthalmologists are not adequately familiar with the signs and symptoms of ocular adnexal and orbital tumors.

The prevalence of ocular adnexal and orbital tumors is different among races. For example, the most common malignant tumor of the eyelids in the USA and Europe is basal cell carcinoma (BCC) which accounts for 80–95% of all eyelid malignancies [1, 2]. Squamous cell carcinomas (SCCs; <5%), sebaceous gland carcinomas (SGCs; 1–3%), malignant melanomas (1%), and miscellaneous tumors (<1%) constitute the remaining eyelid malignancies in the USA and Europe [1, 2]. The reports from Asia are very different. BCC is less common at 11 to 65% while SCC at 5 to 48% and SGC at 7 to 56% occur more frequently than in the Western countries [1, 2]. The results of several studies in Japan indicated that the prevalence of BCC was lower than that in the Western countries. However, there are few epidemiological studies regarding the prevalence and histologically proven types of ocular adnexal and orbital tumors in Japan [3, 4].

The medical records of histopathological examinations are difficult to collect because of several reasons. First, the number of orbital tumors is small. Second, the number of specialists of orbital tumors is also small, and if the specialists move to other hospitals, the patients in that area must be referred to the hospital where he or she has moved. Then it is difficult to assess the long-term progression of the tumor.

In these past 10 years, the Chiba University Hospital had been the main hospital to perform orbital surgeries in Chiba prefecture. Thus, we have collected medical records and histopathological findings of many patients with adnexal and orbital tumors.

It was reported in 2013 that some extranodal marginal zone lymphoma of mucosa-associated lymphoid tissue type (MALT) lymphoma patients were IgG4-positive [5]. We hypothesize that thereafter, the serum levels of IgG4 may be measured more often when MALT lymphoma is suspected and that pathological biopsies may have been done more proactively when IgG4-related disease (IgG4-RD) is suspected from the clinical findings.

Thus, the purpose of this study was to determine the epidemiology of ocular adnexal and orbital tumors that were diagnosed histopathologically in the Chiba University Hospital. In addition, we examine the annual changes in the major types of tumors including malignant lymphomas and IgG4-RDs and discuss the trends and the association between MALT lymphomas and IgG4-RDs.

## Methods

This was a retrospective study conducted at Chiba University Hospital in Japan. A search was conducted of the medical records and ophthalmic pathology database for cases diagnosed with ocular adnexal and orbital tumors. All cases with ocular adnexal and orbital tumors whose diagnosis was based on histopathological examinations during the period from April 2009 to March 2019 were studied. Patients who had multiple surgeries or had taken multiple biopsies and proven to be one specific disease were counted as a single case. In these cases, the lesion site was counted to be the most affected site. Patients with only a clinical diagnosis but without a histopathological examination or patients who only had histopathological examinations were excluded. Thus, intraocular malignant lymphomas were not included because intraocular malignant lymphomas were diagnosed with the combination of cytology and other clinical findings including IL-10/IL-6 ratio.

The following data were extracted from the medical records: age at the first surgery, sex, tumor location(s), initial clinical diagnosis, date of the surgery, treatment details, and final histopathological diagnosis. In addition, the changes in the annual number of patients with major diseases including malignant lymphomas and eyelid tumors were evaluated statistically. Malignant lymphomas, the most frequent malignant tumor, were classified as their different sub-types; MALT lymphomas, diffuse large-B-cell lymphoma (DLBCL), or mantle cell lymphomas. In addition, the number of patients with a MALT lymphoma whose serum IgG4 level was determined was compared between those in the first five years to the last five years of this study.

All of the procedures conformed to the tenets of the World Medical Association Declaration of Helsinki. A written informed consent was obtained from a parent and/or legal guardian for subjects below 18 years. Otherwise, a written informed consent was directly obtained from all participants except for subjects below 18 years. An approval for the study was obtained from the Institutional Review Board of the Graduate School of Medicine, Chiba University, Japan (IRB#3848).

Linear regression analysis was performed to determine the trend of the number of major diseases, i.e., BCC, SGC, malignant lymphoma, IgG4-RD, and MALT lymphoma. Furthermore, linear regression analysis was performed to examine the trend of the ratio of MALT lymphomas among all malignant lymphomas. Data was expressed as the mean ± standard deviation (SD). A value of *P* < 0.05 was considered statistically significant.

## Results

The mean age of all 372 patients was 62.9 ± 18.3 years with a range of newborn to 97 years (Table 1). The mean number of the patients with benign tumors was 221 with a mean age of 59.0 ± 17.9 years, and 151 were malignant with a mean age of 68.9 ± 17.0 years (Table 1). The proportion of benign tumors to all tumors was 59.4% (221/372) and that for malignant tumors was 40.6% (151/372; Table 1). The number of patients with tumors of the conjunctiva or eyelids was 270 and their mean age was 64.1 ± 17.4 years (Table 1). The number of patients with benign tumors was 166 with a mean age of 59.4 ± 18.2 years and that of the malignant tumors was 104 with a mean age of 71.5 ± 13.0 years (Table 1).

**Table 1:** Numbers and distribution of the tumors in eyes and orbit

Location	Number	%	Mean age in years (SD)	Sex (male), n (%)
Conjunctiva/Eyelids	270		64.1 (17.4)	137 (51)
Benign	166	61.50%	59.4 (18.2)	89 (54)
Malignant	104	38.50%	71.5 (13.0)	48 (46)
Orbit	102		59.8 (20.1)	59 (58)
Benign	55	53.90%	57.1 (17.3)	26 (47)
Malignant	47	46.10%	63.0 (22.5)	33 (70)
Total	372		62.9 (18.3)	196 (53)
Benign	221	59.40%	59.0 (17.9)	115 (52)
Malignant	151	40.60%	68.9 (17.0)	81 (54)

*Abbreviation: SD* Standard deviation

The number of patients with orbital tumors was 102 and their mean age was 59.8 ± 20.1 years (Table 1). The number of benign tumors was 55 and the mean age of these patients was 57.1 ± 17.3 years. The remaining 47 were malignant and the mean age of these patients was 63.0 ± 22.5 years (Table 1).

The patients with either malignant conjunctival and eyelid tumors or orbital tumors were significantly older than those with benign tumors (Table 1).

The type of benign tumors diagnosed histopathologically and the numbers and sites, i.e., conjunctival and eyelids:orbital, were; cysts 29 (24:5), nevus 22 (22:0), papilloma 21 (20:1), IgG4-RD 21 (5:21), seborrheic keratosis 19 (19:0), idiopathic orbital inflammatory disease 13 (2:11), hyperplasia 12 (12:0), lymphoid hyperplasia 11 (11:0), pleomorphic adenoma 10 (3:7), hemangioma 9 (7:2), verruca vulgaris 8 (8:0), pyogenic granuloma 3 (3:0), and others 43 (30:13; Table 2). During this period there were 30 non-neoplastic lesions (25 cases of Charaza and 5 cases of Lacrimal canaliculitis), and they are excluded in this study.

**Table 2:** Benign tumors in conjunctiva/eyelids and orbit

Benign					
Tumor	Conjunctiva/Eyelids	Orbit	Total	Mean age in years (SD)	Sex (male), n (%)
Cyst	24	5	29	57.9 (18.1)	17 (59)
Nevus	22	0	22	55.4 (20.7)	7 (32)
Papilloma	20	1	21	53.2 (16.6)	12(57)
IgG4-related disease	5	16	21	62.3 (12.5)	11 (52)
Seborreic keratosis	19	0	19	70.5 (9.6)	11 (58)
Idiopathic orbital inflammatory disease	2	11	13	61.5 (15.2)	6 (46)
Hyperplasia	12	0	12	63.5 (16.6)	8 (67)
Lymphoid hyperplasia	11	0	11	58.1 (14.3)	6 (55)
Pleomorphic adenoma	3	7	10	54.5 (15.4)	5 (50)
Hemangioma	7	2	9	59.8 (19.0)	4 (44)
Verruca vulgaris	8	0	8	72.8 (4.2)	6 (75)
Pyogenic granuloma	3	0	3	69.0 (10.2)	1 (33)
Others	30	13	43		21(49)
Total	166	55	221		

*Abbreviation*. *SD* Standard deviation

Among the malignant tumors, the histopathological diagnosis, and site, i.e., (conjunctival and eyelids:orbital) were; malignant lymphoma 74 (40:34), SGC 28 (28:0), BCC 15 (15:0), SCC 9 (8:1), malignant melanoma 6 (3:3), carcinoma in situ 4 (4: 0), retinoblastoma 4 (0:4), and others 12 (7:5) (Table 3). An additional file shows graphical illustration of the frequencies of major malignant eyelid tumors (see Additional file 1).

**Table 3:** Malignant tumors of conjunctiva/eyelids and orbit

Malignant					
Tumor	Conjunctiva/Eyelids	Orbit	total	Mean age in years (SD)	Sex (male), n (%)
Malignant lymphoma	40	34	74	67.4 (12.3)	38 (51)
Sebaceous gland carcinoma	28	0	28	78.2 (10.8)	12 (43)
Basal cell carcinoma	15	0	15	76.4 (8.2)	6 (40)
Squamous cell carcinoma	8	1	9	71.2 (17.6)	5 (56)
Malignant melanoma	3	3	6	55.7 (8.0)	4 (67)
Carcinoma in situ	4	0	4	74.0 (4.9)	4 (100)
Retinoblastoma	0	4	4	1.0 (0.7)	3 (75)
Others	6	5	11		9 (82)
Total	104	47	151		

*Abbreviation*. *SD* Standard deviation

The malignant lymphomas were divided into MALT lymphomas, diffuse large B-cell lymphomas (DLBCL), mantle cell lymphomas, follicular lymphomas, and B-cell lymphomas. The numbers and the sites (conjunctiva/eyelids:orbit) of each were: MALT lymphomas 51 (30:21), DLBCLs 11 (5:6), mantle cell lymphomas 5 (2:3), follicular lymphomas 4 (3:1), B-cell lymphomas 3 (0:3) (Table 4). B-cell lymphomas were unclassifiable with features intermediate between DLBCLs and classical Hodgkin lymphoma.

**Table 4:** Classification of subtypes of malignant lymphoma

Malgnant Lymphoma					
	Conjunctiva/Eyelids	Orbit	total	Mean age in years (SD)	Sex (male), n (%)
MALT	30	21	51	66.4 (13.7)	26 (51)
DLBCL	5	6	11	71.8 (6.94)	6 (55)
Mantle cell lymphoma	2	3	5	72.6 (3.3)	2 (40)
Follicullar lymphoma	3	1	4	73.8 (8.84)	2 (50)
B-cell lymphoma	0	3	3	69.3 (6.55)	2 (67)
Total	40	34	74	67.4 (12.3)	38 (51)

*Abbreviation*. *MALT* Extranodal marginal zone lymphoma of mucosa-associated lymphoid tissue type, *DLBCL* Diffuse large B-cell lymphoma, *SD* Standard deviation

**Fig. 1:**
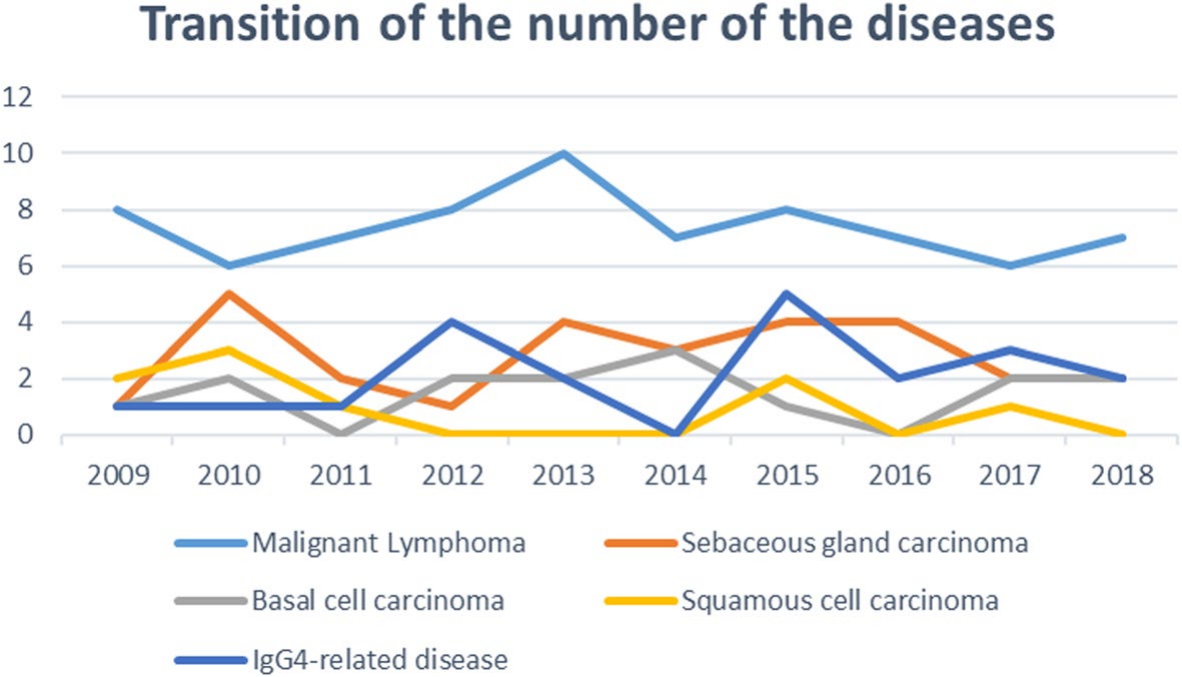
Annual changes of the number of major ocular adnexal and orbital tumors in the last 10 years. Malignant lymphoma is the most common malignant tumor among the ocular tumors in Japan, but the total number of malignant lymphomas does not increase with year by year

**Fig. 2:**
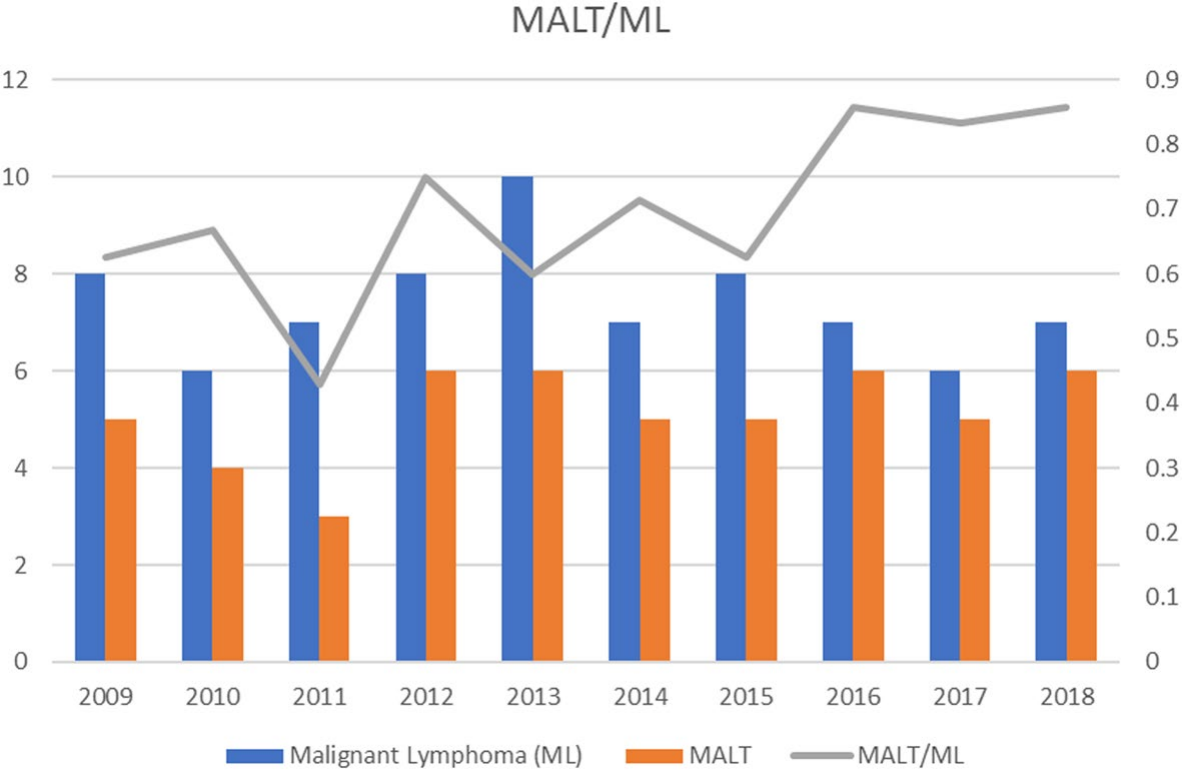
The ratio of MALT lymphoma among all malignant lymphomas in the last 10 years. The ratio of MALT lymphoma in all malignant lymphomas has increased with years (R^2^ = 0.483, *P* = 0.026). MALT; extranodal marginal zone lymphoma of mucosa-associated lymphoid tissue type, ML; malignant lymphoma. Left vertical shows numbers of cases. Right axis shows the ratio (%) of MALT/all ML

**Fig. 3:**
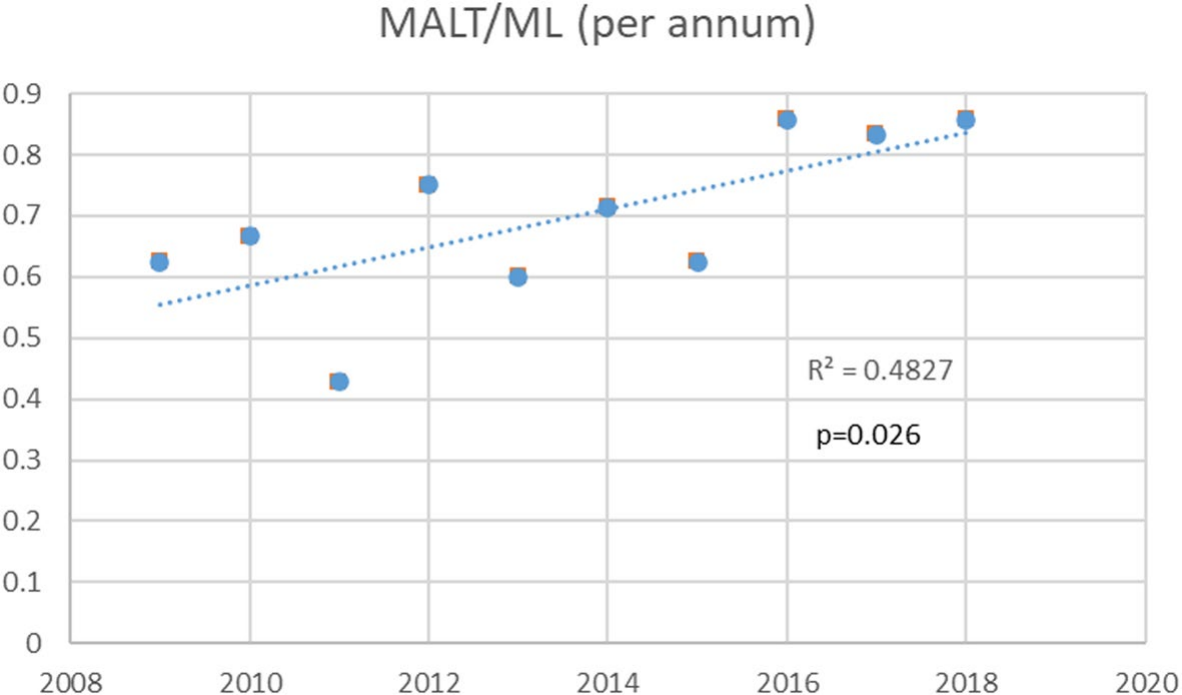
Graph showing the annual changes of the ratio of MALT among all malignant lymphomas. The ratio is increasing with years (R^2^ = 0.483, *P* = 0.026). MALT; extranodal marginal zone lymphoma of mucosa-associated lymphoid tissue type, ML; malignant lymphoma. Vertical numbers show the ratio (%). Horizontal numbers show years

The annual changes in the numbers of malignant lymphomas, SGC, BCC, SCC, and IgG4-related disease, are shown in Fig. 1. The number of all tumors did not increase significantly during the study period as determined by linear regression analysis; malignant lymphomas (R^2^ = 0.035, *P* = 0.60), SGC (R^2^ = 0.0062, *P* = 0.83), BCC (R^2^ = 0.017, *P* = 0.72), SCC (R^2^ = 0.303, *P* = 0.10), and IgG4-RD (R^2^ = 0.122, *P* = 0.32), respectively (Fig. 1). Similarly, the number of MALT lymphomas did not increase with year by year (R^2^ = 0.248, *P* = 0.14; Fig. 2). On the other hand, the ratio of MALT among all malignant lymphomas increased significantly during the observation period by linear regression analysis (*P* = 0.026; Fig. 2). The annual changes of the ratio of MALT among all malignant lymphomas are shown in Fig. 3 (R^2^ = 0.483, *P* = 0.026).

**Fig. 4:**
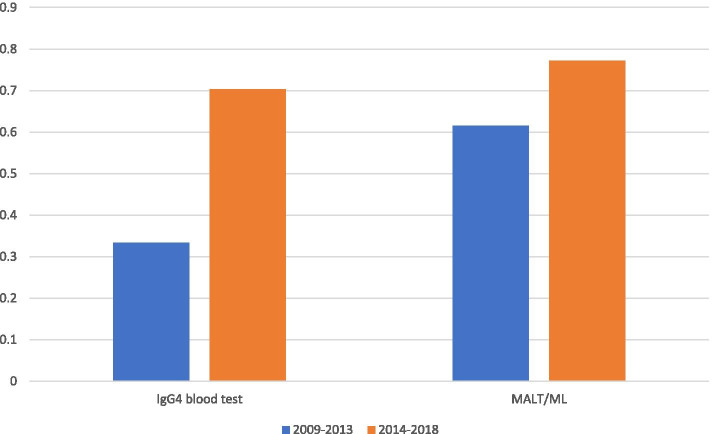
Ratio of patients with MALT lymphoma who underwent IgG4 blood test and the ratio of patients with MALT lymphoma among all types of malignant lymphoma between 2009 and 2013 vs. 2014 and 2018. The ratio of patients who had suspected malignant lymphoma and underwent the serum IgG4 is higher in the last five years than in the first five years (Left bars). The ratio of MALT lymphoma among all malignant lymphoma was greater in the last five years than in the first five years (Right bars)

The ratio of patients with MALT lymphomas who underwent IgG4 blood test and the ratio of patients with MALT lymphoma among all types of malignant lymphoma between 2009 and 2013 vs. 2014 and 2018 are shown in Fig. 4. The ratio of patients with MALT lymphoma who underwent serum IgG4 test or measurement was more in the last five years than in the first five years (Fig. 4). Among them, one patient in the first five years and one patient in the last five years showed IgG4-positive (543 mg/dl and 149 mg/dl, respectively). The ratio of MALT lymphoma among all malignant lymphoma was more in the last five years than in the first five years (Fig. 4).

## Discussion

The percentage of benign cases of all ocular tumors was <60% which is consistent with an earlier report [5]. The possible reason for the relatively low number of benign cases is that our institute is the University Hospital, and mainly suspected malignant cases are usually referred from other clinics and hospitals [6].

Although BCC is the most common malignant tumor of the eyelids in Western counties (80–91%), the percentage of SGC was higher than BCC in our hospital [4, 7, 8]. Several studies on the Japanese population have shown that SGC is as common as BCC, and that SCC is not rare in Japan [4, 9–12]. The results of our study are consistent with these Japanese reports as well as reports from other Asian counties [2, 13–15]. Moreover, SGC was seen more often than BCC and SCC. SGC is characterized by high rate of recurrences and regional and distant metastases. Thus, patients may have been referred to our hospital for more extensive resections after an initial surgery at another hospital [16].

The percentage of pleomorphic adenomas in the Japanese population was reported to be 7% which is higher than the 1.4 to 4.3% reported in the USA and European countries.^3^ However, a percentage of 2.5% was more likely to the latter. Further nation-wide epidemiological studies may be necessary for examining the prevalence of pleomorphic adenomas in Japan.

In the WHO classification, MALT lymphoma is defined as an extranodal lymphoma composed of morphologically heterogeneous small B-cells including marginal zone (centrocyte-like) cells, cells resembling monocytoid cells, small lymphocytes, or scattered immunoblast and centroblast-like cells. There is plasma cell differentiation in some of the cases. The infiltrates are in the marginal zone of reactive B-cell follicles, and extend into the interfollicular region. MALT lymphomas may be acquired secondary to autoimmune diseases or from an infection at a given site providing the substrate for the development of a lymphoma [17].

MALT lymphomas are generally painless and slow-growing, and they usually present as localized extranodal tumors without accompanying adverse prognostic factors. Patients with these tumors have a good prognosis [18–20]. Many worldwide studies have reported that MALT lymphomas are the most common subtype of malignant lymphomas among the ocular and orbital lymphomas (60–80%) [17, 21–23]. However, none of the studies reported that the ratio of MALT among all malignant lymphomas was increasing year by year. It is difficult to demonstrate the increase of the ratio of MALT lymphomas among all malignant lymphomas because in the studies that reported the proportion of MALT lymphoma, the number of the cases was small. Thus, it may be difficult to detect a trend. Another possible reason is the great improvements in the imaging techniques. An increase in the incidence of primary ocular adnexa lymphomas over the last decades has been suggested to be because of the advances in imaging techniques that have enabled clinicians to justify more biopsies. Thus, specimens can be obtained for suitable immunohistochemical staining [24]. In addition, the development of immunohistochemical and molecular techniques has resulted in an increase in the accuracy of the diagnosis of low-grade small B-cell lymphoma which is the most commonly found orbital lymphoma. Small B-cell lymphomas, such as MALT lymphomas, may have been formerly diagnosed as reactive lymphoid hyperplasia, pseudo-lymphoma, atypical lesion or borderline lesion and therefore diagnosed as inflammatory pseudo-tumors [3]. Thus, the proportion of MALT lymphoma may increase as the techniques improve.

MALT lymphomas must be differentially diagnosed from IgG4-RD but it is not easy to perform histopathological examinations for all patients. Therefore, there might have been some cases that were diagnosed as IgG4-RD by only the clinical findings and blood tests. It has been reported that 11% of patients with MALT lymphomas were IgG4-positive, and it is known that there is an increase of the serum IgG4 level in patients with MALT lymphomas [5].

Actually, the ratio of the patients who had taken IgG4 blood tests and had been finally diagnosed with MALT lymphoma was only 33% (8/24) in the first 5 years which then increased to 70% (19/27) in the latter 5 years. In total, two patients were IgG4-positive (7.4%); in the first five years, one patient with MALT lymphoma was IgG4-positive, and in the last five years one patient with MALT lymphoma was IgG4-positive. Also, the ratio of MALT lymphomas among all ML patients had been increasing during this study period. On the other hand, there were no other types of malignant lymphomas that were IgG4-positive.

There may have been some patients with MALT lymphomas who were initially diagnosed as IgG4-RD and treated with steroid because of the increase of serum IgG4 level. However, their condition did not improve and may be regarded as being refractory IgG4-RD. MALT lymphoma is a malignant tumor and a life-threatening disease. Thus, pathological biopsies should be performed even when the serum level of IgG4 is increased.

Most of the malignant lymphoma patients were not treated in our department but in the Hematology Department or at other local hospitals after making the histopathological diagnosis. This makes it difficult to follow the entire course of the disease process. At least 37 of 51 MALT lymphoma cases (72.5%) underwent radiation therapy and 6 cases (11.8%) were treated by chemotherapy. Among 11 DLBCL cases, at least 4 cases (36.4%) underwent radiation therapy and 8 cases (72.7%) received chemotherapy. Likewise, 1 of 5 mantle cell lymphoma cases (20.0%) underwent radiation therapy and no chemotherapy was used, 3 of 4 follicular lymphoma cases (75.0%) received radiation therapy, and 1 of 3 B-cell lymphoma cases (33.3%) received radiation therapy and 2 cases (66.7%) were treated by chemotherapy.

There are some limitations of this study. First, although the number of the cases is not so small considering the incidence of ocular tumors, but it is still too small to detect the trends of each disease. Multicenter studies may be required to gather more data for a stronger analyses and conclusions. Second, this was a retrospective study. Thus, further additional prospective epidemiological studies may be needed. Third, we have Chiba Children’s Hospital in Chiba prefecture and most children with malignant tumors including retinoblastoma may be referred to Chiba Children’s Hospital. In fact, the number of retinoblastomas is fewer than other epidemiological studies.

## Conclusions

The results of this epidemiological study of ocular adnexal and orbital tumors in Japan showed that the frequency of BCC in Japan is less frequent than in Western countries, but SGC was seen much more often. On the other hand, pleomorphic adenoma was seen less than expected. We found that the ratio of MALT lymphoma in all malignant lymphomas has been increasing with years. Pathological biopsy should be done proactively not to miss IgG4 positive MALT lymphomas. Because the small sample size and the retrospective nature of the study, the results of this study should be considered with caution.

## Supplementary Information

**Additional file 1: Supplementary Figure 1.** Graphical illustration of the frequencies of major malignant eyelid tumors.

## Data Availability

The datasets used or analysed during the current study are available from the corresponding author on reasonable request.

## Abbreviations

BCC: Basal cell carcinoma
SCC: Squamous cell carcinoma
SGC: Sebaceous gland carcinoma
MALT: Extranodal marginal zone lymphoma of mucosa-associated lymphoid tissue type
IgG4-RD: IgG4-related disease
DLBCL: Diffuse large-B-cell lymphoma
SD: Standard deviation
