# Supplementary material for: Ten-year epidemiological study of ocular and orbital tumors in Chiba University Hospital
Source: BMC Ophthalmol. 2021 Sep 23;21:344. doi: 10.1186/s12886-021-02108-w (PMC8459513; doi:10.1186/s12886-021-02108-w)

Title:

Ten-year epidemiological study of ocular and orbital tumors in Chiba University Hospital

Authors: Norihiro Shimizu[1], Toshiyuki Oshitari[1,2], Jiro Yotsukura[1], Hirotaka Yokouchi[1], Takayuki Baba[1], Shuichi Yamamoto[1]

Institutes: [1]Department of Ophthalmology and Visual Science, Chiba University Graduate School of Medicine, Chiba, Japan

[2]Department of Ophthalmology, International University of Health and Welfare, Narita, Japan

Corresponding Author: Toshiyuki Oshitari

Department of Ophthalmology and Visual Science, Chiba University Graduate School of Medicine, Inohana 1-8-1, Chuo-ku, Chiba 260-8670, Chiba, Japan.

TEL/FAX: 81-43-226-2124/81-43-224-4162

E-mail: Tarii@aol.com

Supplementary figure 1

Graphical illustration of the frequencies of major malignant eyelid tumors. In Japan, sebaceous gland carcinoma (SGC) is the most common malignant eyelid tumor and basal cell carcinoma (BCC) is the second most malignant tumor.

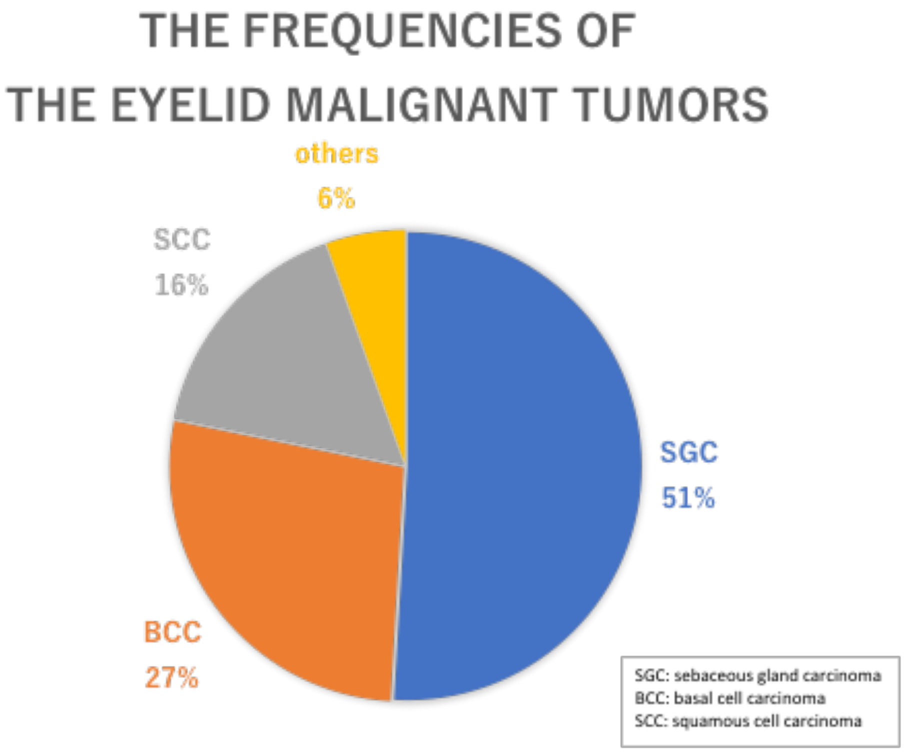

Supplement: Supplementary file 1 — Additional file 1: Supplementary Figure 1. Graphical illustration of the frequencies of major malignant eyelid tumors. [file 12886_2021_2108_MOESM1_ESM.pdf]
